# Efficacy of controlled-release oxycodone for reducing pain due to oral mucositis in nasopharyngeal carcinoma patients treated with concurrent chemoradiotherapy: a prospective clinical trial

**DOI:** 10.1007/s00520-019-4643-5

**Published:** 2019-02-02

**Authors:** Xin Hua, Lin-Min Chen, Qian Zhu, Wen Hu, Chao Lin, Zhi-Qing Long, Wen Wen, Xiao-Qing Sun, Zi-Jian Lu, Qiu-Yan Chen, Dong-Hua Luo, Rui Sun, Hao-Yuan Mo, Lin-Quan Tang, Wen-Wen Zhang, Zhen-Yu He, Hai-Qiang Mai, Huan-Xin Lin, Ling Guo

**Affiliations:** 1 State Key Laboratory of Oncology in South China, Collaborative Innovation Center for Cancer Medicine, Sun Yat-sen University Cancer Center, No. 651, Dongfeng East Road, Guangzhou, 510060 China; 2 Department of Radiotherapy, Sun Yat-sen University Cancer Center, Guangzhou, China; 3 Department of Nasopharyngeal Carcinoma, Sun Yat-sen University Cancer Center, Guangzhou, China

**Keywords:** Nasopharyngeal carcinoma, Concurrent chemoradiotherapy, Oral mucositis, Controlled-release oxycodone, Efficacy

## Abstract

**Background:**

Pain due to oral mucositis (OM) is a major problem during concurrent chemoradiotherapy (CCRT) in nasopharyngeal carcinoma (NPC) patients.

**Methods:**

We enrolled 56 NPC patients receiving CCRT and allocated them into two groups: moderate pain group (*n* = 27) and a severe pain group (*n* = 29) according to the degree of pain reported (moderate = numerical rating scale (NRS) score 4–6 or severe = NRS score 7–10) at initiation of controlled-release oxycodone (CRO) treatment.

**Results:**

Total dose of CRO was significantly higher in severe pain patients than in moderate pain patients (791.60 ± 332.449 mg vs. 587.27 ± 194.940 mg; *P* = 0.015). Moderate pain patients had significantly better quality of life (*P* = 0.037), lower weight loss (*P* = 0.030) and more active CCRT response (90.9% vs. 64.0%; *P* = 0.041). Although 24-h pain control rate was comparable in the two groups (85.2% vs. 86.2%; *P* = 0.508), the moderate pain group score eventually stabilized at ~ 2 vs. 3 in the severe pain group (*P* < 0.001); the titration time to reach bearable pain (NRS ≤ 3) was also significantly shorter in moderate pain patients (2.45 ± 0.60 days vs. 3.60 ± 1.98 days; *P* = 0.012). Incidence of adverse events was comparable in both groups.

**Conclusions:**

The study findings suggest that early introduction of low-dose CRO at the moderate pain stage could help reduce the total dose required, provide better pain control, improve quality of life, and enhance CCRT response.

## Introduction

Nasopharyngeal carcinoma (NPC) is usually (> 70%) diagnosed at a locoregionally advanced stage [1–3], when the standard treatment is concurrent chemoradiotherapy (CCRT) [4]. About 85%–100% of patients receiving chemoradiotherapy for head-and-neck cancer develop oral mucositis (OM) [5–7]. More than 85% of patients have severe OM (WHO grades 3–4) during high-dose radiotherapy [5, 7, 8]. The severity of OM pain is related to the radiation dose, with the mean cumulative dose to reach moderate pain reported to be 24.6 ± 2.0 Gy [9]. OM pain can interfere with eating and adversely affect the quality of life of patients, and may sometimes even be severe enough to cause delay or interruption of treatment [10]. Rapid and sustained relief of pain is therefore essential.

Currently, the primary strategies for preventing or reducing OM pain include nutritional supplementation, oral cleaning, promotion of local mucosal recovery, and use of antibiotics and analgesics [8, 11–13]. Commonly used analgesics are local anesthetic drugs, such as lidocaine mouthwash. However, these measures are not very effective and provide no immediate relief.

According to the WHO Three-Step Treatment of Cancer Pain principles, it is recommended to give short-acting opioids in patients with severe cancer pain to achieve the goal of analgesia and then converted to long-acting opioids maintenance. The National Comprehensive Cancer Network (NCCN) and the European Association for Palliative Care (EAPC) guidelines recommend long-acting opioids (such as oxycodone, fentanyl) as the first-line treatment for cancer pain [14, 15]. While short-acting opioids can provide rapid pain relief, the duration of action is short and multiple doses are necessary for sustained relief [16]. Moreover, titration of dose is complicated and patient compliance poor, resulting in recurrence of pain and even unintentional overdosing [17].

Since 1995, controlled-release oxycodone (CRO) has been widely used for treatment of moderate to severe cancer pain and non-cancer pain [18–21]. In clinical practice, CRO is widely used as the second- or third-step analgesic [22–24]. It is a pure opioid-receptor agonist without a “ceiling effect” [25] and with an analgesic effect that is 1–1.5 times stronger than that of oral morphine. Moreover, the AcroContin™ controlled-release formulation avoids the problem of the “peak–valley” phenomenon [26, 27]. Previous studies have found that CRO can safely and efficiently alleviate the pain of various cancers and improve the quality of life (QOL) of patients [28], but correct time for initiating CRO medication remains unclear. To date, no study has investigated the use of CRO for control of CCRT-induced OM pain in NPC patients. This prospective clinical trial was designed to examine the efficacy of CRO in controlling OM pain in NPC patients receiving CCRT and to determine the appropriate time for initiation of treatment.

## Methods

### Patients

The study population was comprised of NPC patients without distant metastasis receiving CCRT at Sun Yat-Sen University Cancer Center in China between May 19, 2015, and January 23, 2018. The study protocol was approved by the institutional review board of Sun Yat-sen University Cancer Center and written informed consent was obtained from all enrolled patients. The study was registered at Clinical Trails.gov NCT03045484. The key raw data was uploaded onto the Research Data Deposit (RDD) public platform (www.researchdata.org.cn), with the approval RDD number as RDDA2018000731.

### Inclusion and exclusion criteria

First, to evaluate the exact time of introduction of CRO, we conduct a preliminary experiment of 10 patients: 1 patient needed pain intervention at 12F of radiotherapy, 1 at 14F, 5 at 15F, 2 at 16F, and 1 at 17F. Considering the result of the preliminary experiments, the majority of patients (5/10) require pain relief at 15F of radiotherapy; we define 15F as the time node to introduce CRO. Patients were eligible for inclusion if they 1) were aged 18–65 years, 2) had been newly diagnosed with NPC without distant metastasis and were receiving CCRT, 3) need pain intervention and experienced moderate to severe OM pain (numeric rating scale (NRS) score ≥ 4) at 15F of radiotherapy, 4) had not received opioid analgesics previously, 5) were able to understand the application of the NRS for assessing pain level, 6) had normal hematology examination results, and 7) were willing to give written informed consent. Patients were excluded if they 1) refused treatment with CRO, 2) need pain intervention before or after 15F, 3) had only mild oral mucosal pain (NRS score < 4) at 15F, and 4) had any mental illness. The enrolled patients were separated into two groups according to the degree of pain experienced at 15F of radiotherapy (the best pain intervention time node) when CRO was initiated: a moderate pain group (NRS score 4–6) or a severe pain group (NRS score 7–10).

### CCRT protocol

The CCRT schedule comprised 100 mg/m2 cisplatin given intravenously every 3 weeks concurrently with intensity-modulated radiation therapy. Total cumulative doses were > 66 Gy to the primary tumor and > 50 Gy to the bilateral cervical lymph nodes and potential sites of local infiltration. The radiation dose was 2.0–2.27 Gy per fraction with five daily fractions per week for 6–7 weeks, administered as megavoltage photons.

### CRO dose titration

**Fig. 1:**
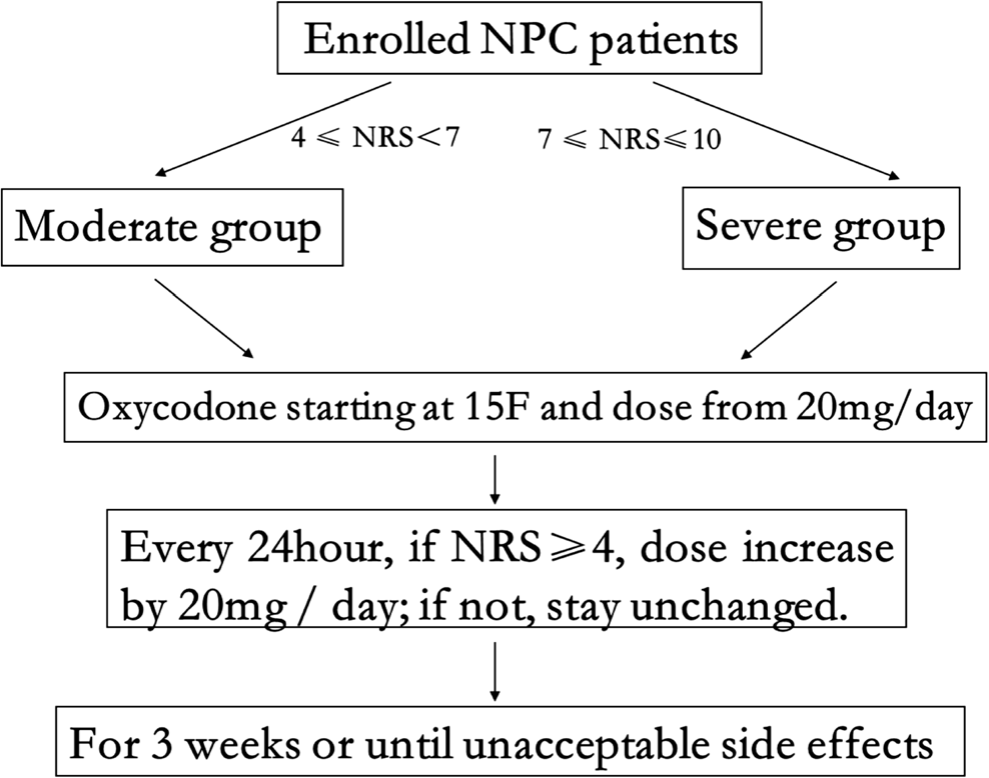
Study design. NPC, nasopharyngeal carcinoma. 15F, the fifteenth fractionated radiotherapy. NRS, numerical rating scale

CRO treatment was initiated with dose of 20 mg/day (10 mg q12h; Fig. 1). Every 24 h, a resident doctor recorded the CRO dose, and patients were asked to indicate the average severity of their pain over the previous 24 h on an 11-point NRS on which the score ranged from 0 (“no pain”) to 10 (“unbearable/severe pain”) [29]. If the NRS score was ≥ 4 and no adverse events were reported, the CRO dose was increased by 20 mg (i.e., another 10 mg q12h). Rescue doses with normal-release morphine and opioid antagonists (e.g., single-ingredient naloxone or naltrexone) were permitted, but they had to be recorded in the patient’s diary. If the NRS score was < 4, the CRO dose was left unchanged. After completion of CCRT, the same procedure was followed in reverse to gradually reduce dose.

### Study assessments

All patients were observed from 15F to 30F of radiotherapy for 3 weeks. Pain was evaluated every day using the NRS scale, weight and nasopharyngoscopic examination were recorded every week; QOL was assessed every 3 weeks using the WHO Quality-of-Life Questionnaire-100 (WHO QOL-100) [30]; and MRIs were conduct at 30F. Degree of pain and any adverse effects were recorded daily in a patient diary and evaluated by a senior doctor every week. Lactulose (Duphalac) or glycerine enema was prescribed when necessary for constipation. Prophylactic antiemetic drugs (5-hydroxytryptamine-3 receptor antagonists such as tropisetron, granisetron, palonosetron) were also prescribed as needed.

The primary efficacy endpoint was the total dose of CRO from baseline to the end of week 3. Secondary endpoints were 1) 24-h pain effective rate (ER), which was defined as pain remission of ≥ 25% over 24 h; the remission rate was calculated using the formula: (predose NRS − NRS after 24 h/predose NRS) × 100%); 2) weight loss from baseline to week 3; and 3) change in QOL from baseline to week 3, as assessed by the WHO QOL-100) plus an additional question (question 101) on appetite in which the responses were scored as follows: 1 = very bad, 2 = bad, 3 = average, 4 = good, 5 = very good); 3) CCRT response based on nasopharyngoscopic examination and MRIs, completely response (CR) defined as the complete resolution of assessable nasopharyngeal primary tumors.

AEs—defined as any undesirable and unintended sign, symptom, or disease temporally and possibly associated with the use of CRO—were graded using the Common Terminology Criteria for Adverse Events v 3.0 [31]. Clinically significant abnormalities in laboratory tests, electrocardiogram, and vital signs were also recorded.

### Statistical analysis

Statistical analysis was performed using SPSS software, version 19.0 (IBM Corp., Armonk, NY, USA). A total of 56 patients received the same treatment. The safety analysis/adverse events analysis was performed on all 56. But 9 patients dropped out of the study for various reasons and so final analysis to establish efficacy of treatment was performed only the data of 47 patients. All analyses were conducted using available data, and no imputation was performed for missing data, except for the analysis of the QOL, where missing data were handled using mean/mode imputation. Student’s *t* test was used for comparison of continuous variables. The chi-square test and Fisher’s exact test were used for categorical variables. The percentage of patients experiencing at least one AE was calculated for each group. All statistical tests were two-sided. *P* ≤ 0.05 was considered statistically significant.

## Results

### Patient characteristics

**Fig. 2:**
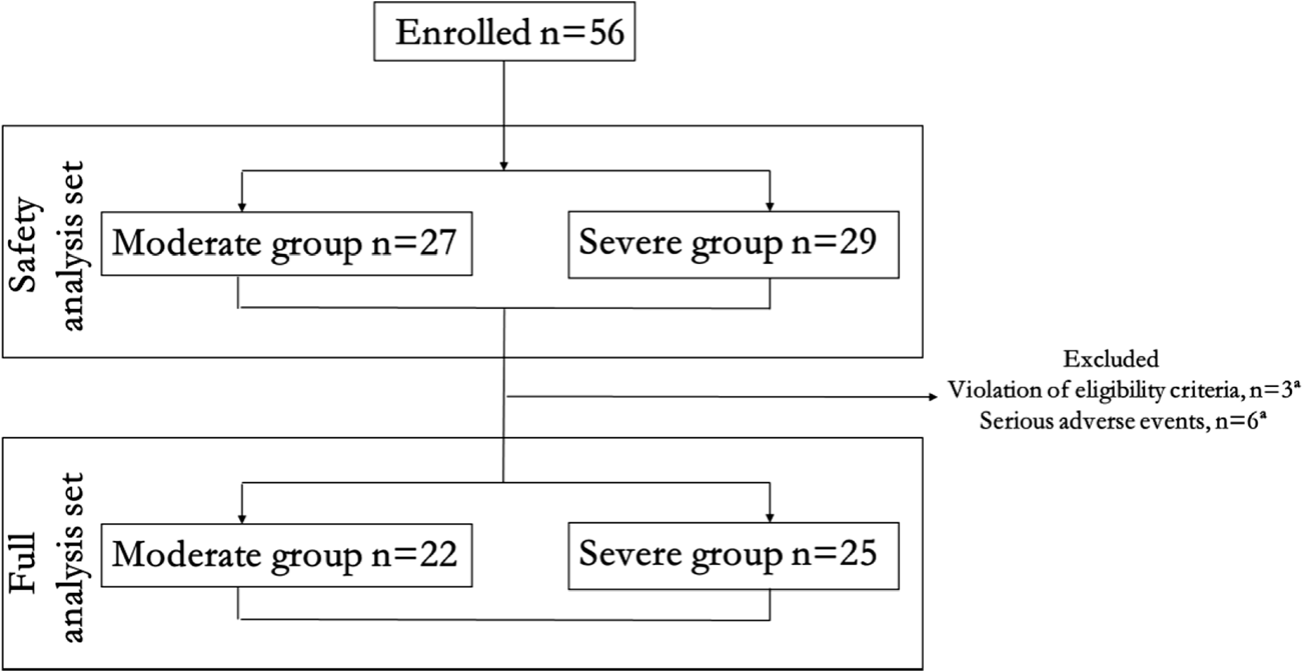
Patient inclusion for safety analysis and full analysis. Three patients were excluded from the final analysis because of violation of the study protocol; these included 2 patients who changed to another analgesic because of poor pain relief with CRO (1 patient in the moderate pain group and 1 patient in the severe pain group) and refusal to take CRO after relief of pain (1 patient in the moderate pain group). Another 6 patients prematurely discontinued the study because of serious adverse events; these included dizziness (1 event), somnolence (1 event), and vomiting (1 event) in the moderate pain group, and dizziness (1 event), vomiting (2 events) in the severe pain group

Figure 2 shows the allocation of patients to the two groups. A total of 56 patients were enrolled in this trial. All 56 patients considered for the safety analysis. However, 9 patients were excluded from the full analysis: 3 because they violated the eligibility criteria (change to another analgesic (*n* = 2), refusal to accept CRO after relief of pain (*n* = 1)) and 6 because they had serious AEs. Thus, for the full analysis, there were only 47 patients (22 in the moderate pain group and 25 in the severe pain group). Table 1 shows the demographics characteristics and clinical characteristics of the study patients at baseline.

**Table 1:** Demographics and clinical characteristics of patients

Variable		Moderate pain group	Severe pain group	*P*
Total (*n*)		27	29	
Age (years) (*n*%)	≥ 60	2 (7.4)	3 (10.3)	0.135†
	< 60	25 (9.6)	26 (89.7)	
Gender (*n*%)	Male	21 (77.8)	26 (89.7)	0.240†
	Female	6 (22.2)	3 (10.3)	
AJCC stage (*n*%)	IV	5 (18.5)	9 (31.0)	0.603‡
	III	21 (77.8)	17 (58.6)	
	II	1 (3.7)	3 (10.3)	
Pain (NRS score) (*n*%)	4	6 (22.2)		NA
	5	5 (18.5)		
	6	16 (59.3)		
	7		16 (55.2)	
	8		11 (37.9)	
	9		1 (3.4)	
	10		1 (3.4)	

NRS scale, numerical rating scale; NA, not applicable; AJCC stage, American Joint Committee on Cancer 7.0

†*P* value calculated with the *t* test

‡*P* value calculated with the Mann-Whitney *U* test

### Total dose

The mean total dose of CRO in the FAS was 695.96 ± 292.63 mg. The mean total dose of CRO was significantly lower in the moderate pain group than in the severe pain group (587.27 ± 194.94 mg vs.791.60 ± 332.45 mg; *P* = 0.015).

### Pain score

**Fig. 3:**
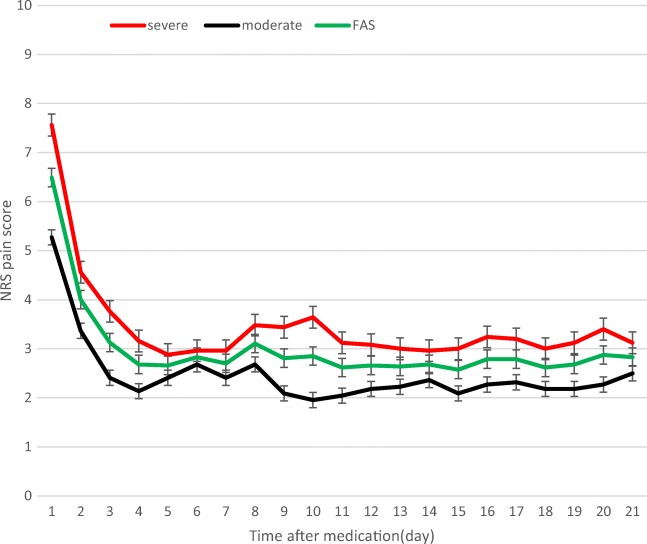
Reduction in NRS pain scores. Reduction in pain scores from baseline to week 3 in the moderate and severe pain groups (full analysis set, FAS). The error bars represent standard deviation (SD). NRS numeric rating scale

Among all 56 patients, the 24-h ER (proportion achieving ≥ 25% reduction in pain) was 85.7% (48/56). There was no significant difference in the 24-h ER between the moderate pain and severe pain groups (85.2% vs. 86.2%; *P* = 0.508). As Fig. 3 shows, there was marked decrease in pain over the first 3 days in the full analysis population, and effective and stable pain control was achieved from day 4 onward. After day 4, the NRS score in the moderate pain group stabilized at ~ 2 points vs. 3 points in the severe pain group (*P* < 0.001). The titration time to achieve bearable pain (NRS ≤ 3) was significantly shorter in the moderate pain group than in the severe pain group (2.45 ± 0.60 days vs. 3.60 ± 1.98 days; *P* = 0.012).

### QOL assessment

Table 2 shows the mean QOL scores. The moderate pain group did not show significant change in QOL scores from baseline to week 3. However, in the severe pain group, there was significant decrease in the QOL variables of “independence” and “pain” between baseline and end of week 3. The overall change in score from baseline to end of week 3 was comparable in the two groups.

**Table 2:** Patient-reported WHO QOL-100 scores at baseline and end of week 3 in the moderate pain and severe pain groups

Variable	Score at baseline		*P*	Score at week3		*P*	Score change from baseline to week3				*P*
	Moderate pain group	Severe pain group		Moderate pain group	Severe pain group		Moderate pain group	*P*ª	Severe group	*P*ª	
Total score	85.22 ± 10.99	79.89 ± 7.15	0.052	85.26 ± 13.65	78.15 ± 7.33	*0.037*	0.04 ± 9.51	0.984	−1.74 ± 4.61	0.071	0.430
Physical	13.41 ± 1.99	10.08 ± 2.57	*0.000*	13.45 ± 2.71	11.47 ± 2.12	*0.007*	0.045 ± 1.55	0.892	−0.30 ± 1.28	0.259	0.262
Pain	11.86 ± 2.32	14.00 ± 2.77	*0.007*	10.73 ± 3.36	12.44 ± 2.47	*0.050*	−1.14 ± 3.00	0.090	−1.56 ± 2.52	*0.050*	0.601
Energy	13.41 ± 2.38	11.04 ± 2.81	*0.003*	12.68 ± 2.85	11.24 ± 2.22	0.058	−0.73 ± 2.03	0.107	0.20 ± 1.92	0.606	0.114
Sleep	14.68 ± 2.90	11.36 ± 3.34	*0.001*	14.41 ± 2.68	11.60 ± 2.40	*0.000*	−0.27 ± 2.43	0.605	0.24 ± 3.03	0.696	0.530
Psychological	15.01 ± 2.22	14.10 ± 1.45	0.101	14.65 ± 2.41	13.81 ± 1.34	0.153	−0.36 ± 1.68	0.332	−0.30 ± 1.28	0.259	0.893
Independence	15.26 ± 2.17	14.25 ± 1.67	0.078	14.98 ± 3.01	13.38 ± 1.94	*0.040*	−0.28 ± 2.24	0.559	−0.87 ± 1.36	*0.004*	0.278
Social	15.38 ± 1.96	14.73 ± 1.38	0.195	15.39 ± 2.11	14.41 ± 1.44	0.067	0.02 ± 1.60	0.965	−0.32 ± 1.29	0.225	0.431
Environment	14.16 ± 2.51	12.93 ± 1.65	*0.049*	14.28 ± 2.59	12.89 ± 1.50	*0.026*	0.12 ± 2.01	0.783	−0.04 ± 1.12	0.860	0.734
Spirit	12.00 ± 2.91	13.08 ± 3.30	0.244	12.50 ± 3.53	12.20 ± 2.45	0.734	0.50 ± 3.28	0.482	−0.88 ± 2.35	0.073	0.101
General health condition	14.32 ± 2.90	13.04 ± 2.09	0.087	15.09 ± 2.84	13.68 ± 2.16	0.060	0.77 ± 2.65	0.186	0.64 ± 1.98	0.119	0.846

WHO QOL-100: The self-administered WHO Quality-of-Life Questionnaire-100. Results are presented as mean ± standard deviation. *P* value: difference between moderate and severe group. *P*ªvalue: difference between baseline score or week 3 score in moderate group or severe group. Significant results are in italics

### Appetite and weight loss

The appetite score was significantly better in the moderate pain group than in the severe pain group at baseline (3.18 ± 0.91 vs. 2.64 ± 0.64; *P* = 0.021) and at week 3 (3.14 ± 1.25 vs. 2.40 ± 0.87; *P* = 0.022). Mean weight loss from baseline to week 3 in the full analysis population was 4.11 ± 2.64 kg. Weight loss was significantly lower in the moderate pain group than in the severe pain group (3.23 ± 2.05 kg vs. 4.88 ± 2.90 kg; *P* = 0.030).

### CCRT response

We compare the CCRT treatment effect of the two group at 30F according the nasopharyngoscopic and MRI examination. Mean CR (complete response) rate in the full analysis population was 76.6% (36/47). CR rate was significantly higher in the moderate pain group than in the severe pain group (90.9% (20/22) vs. 64.0% (16/25); *P* = 0.041).

### Safety

The two groups were comparable with respect to the occurrence rates of any AEs (*P* > 0.99) and serious AEs (*P* > 0.99). During the study, 23/27 (85.2%) moderate pain group patients experienced 31 AEs, and 25/29 (86.2%) severe pain group patients experienced 33 AEs (Table 3). A total of 6 patients (3 in the moderate pain group and 3 in the severe pain group) prematurely discontinued the study because of serious AEs; these included dizziness (1 event), somnolence (1 event), and vomiting (1 event) in the moderate pain group, and dizziness (1 event) and vomiting (2 events) in the severe pain group. Night outbreak of pain requiring additional morphine for pain relief occurred in 2 patients in the severe pain group (in 1 patient on day 8 and in the other on day 10). None of the patients had clinically significant abnormalities in laboratory test results, electrocardiograms, or vital signs.

**Table 3:** Summary of patients in the moderate and severe groups who experienced adverse events (AEs) during the study (safety analysis population)

Adverse event		Moderate pain group (events%)	Severe pain group (events%)	*P*
Total		31 (100)	33 (100)	1.000†
Constipation	Grade I	9 (29.0)	8 (24.2)	0.708‡
	Grade II	6 (19.4)	6 (18.2)	
	Grade III	2 (6.5)	3 (9.1)	
Vomiting	Grade I	5 (16.1)	5 (15.2)	0.722‡
	Grade II	3 (9.7)	4 (12.1)	
	Grade III	1 (3.2)	1 (3.0)	
	Grade IV	1 (3.2)	2 (6.1)	
Dizziness		1 (3.2)	2 (6.1)	1.000†
Somnolence		2 (6.5)	1 (3.0)	1.000†
Dysuria		1 (3.2)	1 (3.0)	1.000†

†*P* value calculated with the continuous correction chi-square test

‡*P* value calculated with the Mann-Whitney *U* test

## Discussion

Radiation therapy to the nasopharyngeal and bilateral cervical lymphatic drainage areas inevitably irradiates the mouth, oropharynx, and hypopharynx [32], and results in dose-dependent oral and pharyngeal mucosal inflammation and pain that may sometimes even be severe enough to require discontinuation of therapy. Currently available treatments have poor efficacy. This study aimed to evaluate the efficacy of CRO in CCRT-induced OM pain in NPC. At present, there is not any kind of good method to control the OM pain, commonly used analgesics are local anesthetic drugs, such as lidocaine mouthwash. It has been reported that Chinese shuanghuabaihe tablets can reduce the occurrence and severity of OM of NPC patients undergoing CCRT [13]. Similarly, kangfuxin solution also reported acceptable for clinical application in NPC patients [12]. But their pharmacological function remains unclear and their efficacy and security are lacking in large sample validation. CRO is a long-acting opioid used as first-line oral opioids for cancer pain for many years [18, 19, 27]. As early as 1997, CRO has been effectively and safely used to treat cancer pain and was an effective alternative to oral morphine [19]. Ferraese et al. reported that CRO can rapidly and effectively manage moderate to severe cancer pain with minimum side-effects [33]. Current reports suggest that oxycodone offers similar levels of pain relief and overall adverse events to other strong opioids including morphine [34]. H. Takase et al. reported the effective use of CRO for the treatment of OM pain caused by radiotherapy in head and neck cancer [9].

In this study, the total dose of CRO was significantly lower in moderate pain patients than in severe pain patients. Thus, the time to reach the peak dose is reduced, as also the time to reduce dose after treatment completion. This can result in a considerable cost savings (as CRO formulations are expensive) and also greatly increase patient compliance with treatment.

In our sample, the NRS score in the moderate pain group remained significantly lower than that in the severe pain group. Furthermore, the 24-h ER was higher and the titration time to stable pain control less in the moderate pain group. Rapid, stable, and effective pain control will lead to increased food intake and decreased weight loss in NPC patients during CCRT and, overall, improve the QOL of patients.

According to clinical practice, OM pain can interfere with eating and adversely affect the quality of life of patients [35], and may sometimes even be severe enough to cause delay or interruption of treatment [10], which are bound to affect the overall efficacy of CCRT. In fact, our analysis demonstrated there is no one delay or interruption of CCRT, all enrolled patients completed the full course of CCRT, with sufficient doses and intensity. And there were significantly better QOL (especially in relation to pain, energy, and sleep) and lower weight loss in the moderate pain group than in the severe pain group. That is, an early introduction of CRO at 15F can rapid and sustained relieve the OM pain to ensure a full course of CCRT. Previous reports from our cancer center have shown that better QOL and maintenance of weight during treatment can improve overall survival and reduce the incidence of distant metastasis in NPC patients [36–38]. Similarly, our research confirms that a better CCRT short-term outcome in the moderate pain group than in the severe pain group, the long-term prognosis still needs further observation. To sum up, early introduction of CRO can help reduce the dose necessary to achieve pain control, achieve better pain control, improve QOL, decrease weight loss, improve compliance with treatment, and ensure better overall effect of CCRT treatment in NPC patients.

The main factor countering the benefits of early introduction of CRO is the probability of adverse events such as constipation, vomiting, anorexia, dizziness, and nausea [25]. Generally, in conformance with the WHO three-step analgesic ladder, the dose of oxycodone is increased in three steps, with the starting dose being 20–40 mg [39]. To reduce the possibility of adverse events, we introduced CRO at a dose of 20 mg and increased the dose gradually. The dose required in the moderate pain group was lower than that in the severe pain group, and so early initiation of CRO could be safer and more cost-effective for the patient. Our study suggests that the early introduction of CRO does not significantly increase the occurrence rate of AEs. There was only one case of drug-withdrawal symptoms but that was in a patient with prior history of drug addiction. The most common AEs in our sample were constipation and vomiting; these were both expected and preventable.

Our trail results revealed that early induction of CRO at the moderate pain had better analgesic qualities and more active CCRT response of NPC patients. The results provide a promising way to guide treatment strategy for OM of NPC patients. Our study has several strengths. First, this is the first time to confirm the efficacy and rational medication time of CRO for OM in NPC patients. Second, we first found that CRO may promote better overall effect of CCRT treatment in NPC patients.

Due to ethical reasons (for patients requiring pain relief interventions, not use CRO treatment is unethical), this experiment was designed as an uncontrolled experiment. Besides above, this study has some limitations. First, this was a single-center trial, and the sample size was small, therefore, we are still proceeding the trail and seeking help from other centers. Second, because of the short duration of observation, we do not have information on long-term prognosis and the occurrence of late AEs; in view of this, we decided to make a long-time follow-up of 2 to 5 years hoping to get a comprehensive prognosis data. Despite these limitations, our study throws some light on strategies for control of OM pain in NPC patients.

## Conclusions

Early introduction of CRO at the moderate pain stage may help reduce the total dose of CRO required, provide better pain relief, reduce weight loss during CCRT, improve the quality of life, and eventually enhance overall effect of CCRT in NPC patients.
